# Development of a practical guide for the early recognition for malignant melanoma of the foot and nail unit

**DOI:** 10.1186/1757-1146-3-22

**Published:** 2010-09-28

**Authors:** Ivan R Bristow, David AR de Berker

**Affiliations:** 1 School of Health Sciences, University of Southampton, Highfield, Southampton, SO17 1BJ, UK; 2 Bristol Dermatology Centre, Bristol Royal Infirmary, Bristol, BS2 8HW, UK

## Abstract

**Background:**

Malignant melanoma is a rare but potentially lethal form of cancer which may arise on the foot. Evidence suggests that due to misdiagnosis and later recognition, foot melanoma has a poorer prognosis than cutaneous melanoma elsewhere.

**Methods:**

A panel of experts representing podiatry and dermatologists with a special interest in skin oncology was assembled to review the literature and clinical evidence to develop a clinical guide for the early recognition of plantar and nail unit melanoma.

**Results:**

A systematic review of the literature revealed little high quality data to inform the guide. However a significant number of case reports and series were available for analysis. From these, the salient features were collated and summarised into the guide. Based on these features a new acronym "CUBED" for foot melanoma was drafted and incorporated in the guide.

**Conclusions:**

The use of this guide may help clinicians in their assessment of suspicious lesions on the foot (including the nail unit). Earlier detection of suspicious pedal lesions may facilitate earlier referral for expert assessment and definitive diagnosis. The guide is currently being field tested amongst practitioners.

## Introduction

The incidence of malignant melanoma (MM) continues to rise in the UK and Europe [1]. Despite being an uncommon form of skin cancer it is responsible for the majority of skin cancer deaths [2]. Health education campaigns have increased public awareness of the problem and there is evidence to show that sectors of the population are presenting earlier with their suspicious skin lesions [3-5]. Despite these improvements, mortality and morbidity still remains high, particularly within subsets of the population such as older adults [6,7], males [8-10], the less affluent [11] and less well educated [12,13].

Around 3-15% of all cutaneous MM arise on the foot [14,15]. However, MM arising on the foot holds a poorer prognosis than melanoma elsewhere [16,17]. The reasons for this are not certain, but there are several possible explanations. The basic prognostic indicator for melanoma at all sites is the thickness of the tumour as measured under the microscope in millimetres. This is known as the Breslow thickness. The greater the thickness of the tumour, the more likely that the patient will die in the following five years. Thick lesions on the hands and feet have been shown to have a worse prognosis than tumours of a similar thickness elsewhere [18]. Some investigators have attributed this to pedal lesions being more aggressive in nature [19], though others have disputed this as a statistical anomaly due to the small numbers involved [20]. Other authors have suggested that the Breslow thickness grading in plantar and nail melanoma is often more difficult to determine or inconclusive [21].

Delay in diagnosis is a further factor, where the length of history of the melanoma has a correlation with Breslow thickness and hence deterioration of prognosis. Foot lesions are often detected by health care practitioners later than lesions elsewhere. A tumour on the face is more likely to result in prompt action by the patient and practitioner than one on the foot. Furthermore, lesions between the toes, beneath the nail or on the sole are further concealed. As a result, delayed presentation results in thicker, more advanced tumours [22-24]. Secondly, foot melanoma, possibly because of its rarity, is frequently misdiagnosed as a more common foot disorder such as tinea pedis [25-29], onychomycosis [30], warts [31-36], haematoma [25,37-39], paronychia [40], ingrowing toe nail [41-43], bacterial infection [44], ischaemia or necrosis [14,40], blisters, ganglions, callus [42], benign tumours [45,46] and ulceration [47-54]. Misdiagnosis rate for foot lesions have been reported to be between 25%-66% [14,25,40] compared with much lower rates of around 12-16% for melanoma in other anatomical locations [27,55,56]. This is probably a reflection of the fact that patients do not initially suspect the diagnosis of skin cancer at these sites and therefore consult healthcare professionals other than dermatologists with lesions who may not be so aware of the possibility of a malignant lesion.

The priority of skin cancer has been highlighted by the Government in its strategy to reform Cancer Services in the UK [57]. Through the "SUNSMART" campaign http://www.sunsmart.org.uk, the government aims to increase public awareness of the disease and stress the importance of seeking professional opinion. On the professional side, guidelines issued from the National Institute for Clinical Excellence (NICE) stress the importance of health care professionals being aware of the modified 7-point checklist [58] for assessment of pigmented skin lesions and where any patient presenting with a skin lesions should be referred to a specialist skin care team [59]. Some guidelines have been published in the UK and Australasia, specifically for medical practitioners for melanoma [60-62] but none are known to exist specifically for lesions arising on the foot. A review of cases in one district [25], demonstrated a significant number of melanoma cases were seen by foot specialists prior to diagnosis.

The need for greater awareness to permit earlier recognition of foot melanoma amongst health care practitioners has been expressed [22]. In turn this could lead to faster recognition, referral and diagnosis. Authors have commented that the traditional melanoma screening algorithms, the ABCDE system & 7-point checklist may be less effective when applied to the foot [25,40,63]. The plantar surface with its thickened epidermis is subject to trauma and hyperkeratotic changes which are not found elsewhere and may disguise critical signs.

In conjunction with the Society of Chiropodists and Podiatrists (Faculty of Podiatric Medicine and General Practice), a panel was convened to draft guidance for its members to raise awareness of the condition. The guide development group consisted of a team consisting of a podiatrist and four dermatologists each with a special interest in skin cancer.

## Methods

Initially the panel compiled a list of clinical questions relevant to the topic of foot and nail melanoma to help inform a search strategy. A literature search was undertaken using the National Library of Medicine (NLM) PubMed database to identify literature on foot and nail melanoma. A range of search terms was devised (see below):

1: foot OR feet OR "lower extremity" OR acral OR plantar OR nail OR leg OR ankle OR sub-ungual (233864)

2: melanoma (74768)

3: Diagn* OR recogn*OR screen* (2518063)

Limits: English Language & Human

Total of combination: 843

From the initial sweep (n = 843), papers whose primary focus fell outside of the topic (i.e. did not discuss recognition, detection, diagnosis and features) were discarded, typically these included papers solely discussing prognosis and survival, surgery and management. Papers which also made brief mention of the foot with no subset analyses were excluded. The remaining papers were reviewed, by both authors, using guidance as outlined by the National Institute for Clinical Excellence [64](Table 1). Papers were classified according to their level of evidence and reviewed for content. In addition, a separate search was undertaken to establish if previous, relevant guidelines had been published elsewhere.

**Table 1:** Levels of evidence (adapted from [64])

Level of Evidence	Type of evidence
1**	High quality meta-analyses, or systematic reviews of randomised controlled trials (RCTs).
1*	Well conducted meta-analyses, systematic reviews of RCTs.
1-	Meta-analyses, systematic reviews of RCTs or RCTs with a high risk of bias.
2**	High quality systematic reviews of cohort or case-control studies. High quality cohort or case-control studies with a low risk of confounding bias or chance.
2*	Well conducted case-control or cohort studies with a low risk of confounding bias or chance.
2	Case-control or cohort studies with a high risk of confounding bias or chance.
3	Non-analytic studies (case reports, case series).
4	Expert opinion, formal consensus.

## Results

The review of the literature identified a lack of high level evidence to inform the development of a guide. Based on the NICE grading system, a small number of case-control studies were identified examining aetiology, incidence and clinical features (level 2). Most of the published literature pertaining to foot melanoma was ranked at level 3, being predominantly case reports (n = 44), literature reviews/discussions (n = 21) and case series (n = 14) of foot melanoma. On this basis, it was accepted that the paper would be drafted on the strength of the available evidence with informed consensus methods amongst the group to develop guidance.

All case reports and case series were examined by the authors. The hierarchy of evidence places such literature at a low level, just above that of medical opinion. However, case studies have the capacity to report rare diseases or the manifestations of disease which can be a useful learning tool in medical education [65]. The authors reviewed these papers looking for common themes, key messages and learning points. The focus of such papers was often around misdiagnosis, delay and deterioration of the lesion. Based on this data, a new acronym was proposed specific for foot melanoma. An existing ABCDE acronym was included for nail melanoma [66].

Subsequent to drafting the paper was reviewed by the panel. External reviewers were identified. These included practising podiatrists and chiropodists, a general practitioner, a diabetologist and other specialists involved in foot care. To facilitate a simple and rapid feedback mechanism, participants were electronically e-mailed a copy of the draft guidelines and then asked to respond by an online feedback website. Respondents were asked to comment on the draft including content, readability and clarity of the draft document. Following the consultation, amendments were made and the guidelines have been reviewed and have been submitted for publication.

## Conclusions

The development and use of a guide may help clinicians in their assessment of suspicious lesions on the foot (including the nail unit). Earlier detection of suspicious pedal lesions may facilitate earlier referral for expert assessment and definitive diagnosis. The guide has been tested amongst practitioners and has been submitted for publication.

## Competing interests

The authors declare that they have no competing interests.

## Authors' contributions

IB was responsible for the original drafting of this paper. Subsequent revisions and amendments were made jointly by DB and IB. Both authors have read and approved the final manuscript.
